# Treatment of oral chronic graft-versus-host disease: a retrospective cohort study

**DOI:** 10.31744/einstein_journal/2021AO6177

**Published:** 2021-10-20

**Authors:** Gabriela de Assis Ramos, Taísa Domingues Boehmer Leite, Camila Brandão Lobo, Paulo Sérgio da Silva Santos, Maria Claudia Rodrigues Moreira, Héliton Spindola Antunes

**Affiliations:** 1 Divisão de Pesquisa Clínica Instituto Nacional de Câncer Rio de JaneiroRJ Brazil Divisão de Pesquisa Clínica, Instituto Nacional de Câncer - INCA, Rio de Janeiro, RJ, Brazil.; 2 Faculdade de Odontologia de Bauru Universidade de São Paulo BauruSP Brazil Faculdade de Odontologia de Bauru, Universidade de São Paulo, Bauru, SP, Brazil.; 3 Centro de Transplante de Medula Óssea Instituto Nacional de Câncer Rio de JaneiroRJ Brazil Centro de Transplante de Medula Óssea, Instituto Nacional de Câncer - INCA, Rio de Janeiro, RJ, Brazil.

**Keywords:** Graft *vs* host disease, Bone morrow transplantation, Hematopoietic stem cell transplantation, Dexamethasone, Tacrolimus

## Abstract

**Objective:**

The aim of this study was to evaluate patients with complete response of oral chronic graft-*versus*-host disease to immunosuppressive treatment.

**Methods:**

A total of 29 patients submitted to allogeneic hematopoietic stem cell transplantation, with oral chronic graft-*versus*-host disease, were enrolled in this retrospective study, from September 2012 to February 2018. Patients were treated with combined topical dexamethasone solution and topical tacrolimus ointment, combined topical dexamethasone and topical tacrolimus, systemic immunosuppressive medication, and topical dexamethasone only.

**Results:**

The mean time of complete response of lichenoid lesions, erythema, and ulcers using dexamethasone and systemic immunosuppressive medication was of 105, 42 and 42 days, respectively (p=0.013).When we associated dexamethasone, tacrolimus and systemic immunosuppressive medication, the mean time of complete response of lichenoid lesions, erythema and ulcers was of 91,84 and 77 days (p=0.011). When dexamethasone was used alone, the mean time of complete response of lichenoid lesions, erythema and ulcers was 182, 140, 21 days, respectively (p=0.042).

**Conclusion:**

Our study shows that lichenoid lesions require more time to heal. Notably, lichenoid lesions tend to respond better to dexamethasone combined with tacrolimus and systemic immunosuppressive medication, whereas erythema and ulcers respond better to dexamethasone combined with systemic immunosuppressive medication and dexamethasone only, respectively.

## INTRODUCTION

Chronic graft-*versus*-host disease (cGvHD) is one of the main complications of hematopoietic stem cell transplantation (HSCT). This condition affects 30% to 70% of patients, leading to inflammatory responses in different organs and tissues that look like autoimmune diseases.^(1)^ In the oral cavity, the clinical symptoms of cGvHD are mainly related to the mucosa and tongue, and are characterized by the presence of lichenoid lesions in the oral mucosa (white streaks), which are recognized by the National Institutes of Health (NIH) consensus as diagnostic lesions. Patients may also present distinctive clinical manifestations that are not sufficient to establish the diagnosis when they occur isolated, such as xerostomia, mucocele, mucosal atrophy, pseudomembranes and ulcers, and clinical manifestations common to acute and cGvHD such as gingivitis, erythema, ulcers, and pain.^(1-4)^

The last NIH consensus^(5)^ recommends topical treatment with high-potency corticosteroids, calcineurin inhibitors and analgesics.^(6,7)^ Local topical medicines have the benefit of intensifying treatment, reducing or eliminating symptoms without increasing systemic immunosuppression.^(8,9)^ Topical treatments may provide better local benefits than systemic therapy alone.^(5)^

Despite the absence of strong evidence, some studies show that most patients with oral cGvHD taking dexamethasone combined with tacrolimus respond positively to treatment.^(9,10)^ In the present observational study, we sought to assess patients with complete response to treatment with topical immunosuppression with oral cGvHD, associated or not with systemic immunosuppression.

## OBJECTIVE

To evaluate patients with complete response of oral chronic graft-*versus*-host disease to immunosuppressive treatment.

## METHODS

### Patients

This retrospective cohort study included patients submitted to allogeneic HSCT, from September 2012 to February 2018, enrolled in the Brazilian *Instituto Nacional do Câncer* (INCA). Eligible patients included men and women aged ≥10 years, who signed and informed consent form, who underwent allogeneic HSCT with a diagnosis of oral cGvHD (lichenoid lesions, erythematous lesions and ulcers) and submitted to topical treatment with dexamethasone with or without tacrolimus, with or without systemic immunosuppression, according to hospital routine. Patients who missed consultation, patients with incomplete response, and patients diagnosed with a second malignant primary tumor in the oral cavity, oropharynx, nasopharynx, hypopharynx, larynx and major salivary glands were considered ineligible. Data were collected from medical and dental records. The study was approved by the Research Ethics Committee protocol number: 4606137; CAAE: 04066512.1.1001.5274.

### Topical treatment for oral chronic graft-*versus*-host disease

Patients included in the study used dexamethasone 0.1mg/mL mouthwash, as first-line treatment, for three minutes, four times a day, after their oral hygiene. Application of 0.1% tacrolimus ointment to the lesion site within the oral cavity was performed twice a day.^(11)^ The option for topical treatment was according to the professional’s individual decision based on severity of the disease and availability of medication.

### Systemic treatment for chronic graft-*versus*-host disease

Patients included in the study were taking the following systemic immunosuppressive medication: prednisone (PDN), ciclosporin (CSA), tacrolimus, methotrexate (MTX) and mycophenolatomofetil (MFM), alone or in combination.

### Assessment of salivary flow and pH

Considering the high frequency of impaired salivary glands in patients with oral cGvHD, with implications for the quality and quantity of saliva,^(12)^ non-stimulated sialometry was performed in patients submitted to HSCT, during follow-up consultation (dental routine of our center). A salivary flow below 0.1mL/minutes was considered hyposalivation.^(13)^ To assess salivary pH were used Color Card MQuant^®^ pH-indicator strips Universal indicator from the Merck Company.

### Assessment of oral chronic graft-*versus*-host disease

In routine care, patients were submitted to dental clinical examination every 21 days, from diagnosis of oral cGvHD to total regression of lichenoid lesions, erythema and ulcers. Severe cases were followed up weekly. Complete response in mouth indicates resolution of reversible clinical manifestations related to oral cGvHD.

We did not use the NIH Oral Mucosa Rating Scale to assess the clinical response, because we did not want an overall assessment of the mouth, but a specific assessment of erythema, ulcers and lichenoid lesions. The following data were collected: the location (right and left oral mucosa, vermilion of the lower and upper lip, upper and lower lip mucosa, right and left lateral border of the tongue, ventral and dorsal tongue, soft palate and hard palate) and duration of oral cGvHD, topical treatment used (dexamethasone rinse and tacrolimus ointment), treatment time and systemic immunosuppressive treatment used.

### Evaluation of tacrolimus serum levels

Serum tests were performed routinely in all patients treated with systemic tacrolimus from diagnosis. However, the assessment of absorption after intraoral topical treatment was not performed due to the lack of standardization of the day and time of collection.

### Statistical analysis

The endpoint of the study was to evaluate oral cGvHD with complete response to topical treatment with dexamethasone, with or without tacrolimus, with or without systemic immunosuppression, through a convenience sample. For discrete variables (types of lesions, lesion locations and drugs used), we evaluated simple frequencies and percentages. For continuous variables, we used medians. The non-parametric Kruskal-Wallis test was used to compare the healing time during the treatment (days) by type of lesion and drugs used and, when significant, multiple comparisons were made to assess differences.

All analyses were performed with SPSS software, version 20.0, considering a significance level of 5% of probability. P values less than or equal to 0.05 were considered statistically significant.

Data from patients with loss of follow-up and who could not be assessed for medication use and treatment response were not included in the assessment.

## RESULTS

This study collected data from patient medical records to evaluate the complete response of oral cGvHD to dexamethasone, with or without tacrolimus, with or without systemic immunosuppression. A total of 76 patients with oral cGvHD were screened, but since it was an observational study, we had to exclude 45 patients for lacking data due to missing consultation. Twenty-nine patients were included and the aim was to evaluate patients with complete response; hence, two patients were excluded because they had lesions with partial response that were considered non-resolved.

The most common underlying disease was acute myeloid leukemia (34.48%), followed by acute lymphoid leukemia (31.03%). Most donors were related (82.75%) and the most used conditioning regimen was cyclophosphamide + busulfan (48.28%) (Table 1).

**Table 1:** Demographic and clinical characteristics

Demographic data	n (%)
Sex	
Male	16 (55.2)
Female	13 (44.8)
Underlying disease	
Acute myeloid leukemia	10 (34.48)
Acute lymphocytic leukemia	9 (31.03)
Chronic myeloid leukemia	4 (13.79)
Myelodysplastic syndrome	3 (10.34)
Aplastic anemia	2 (6.89)
Non-Hodgkin lymphoma	1 (3.44)
Donor	
Related	24 (82.75)
Not related	5 (17.25)
Conditioning regimen	
Cyclophosphamide + Busulfan	14 (48.28)
Cyclophosphamide + TBI + ATG	3 (10.34)
Busulfan + Fludarabine	3 (10.34)
Cyclophosphamide + TBI	3 (10.34)
Cyclophosphamide + Fludarabine	1 (3.45)
Busulfan + Melphalan	1 (3.45)
Cyclophosphamide	1 (3.45)
Busulfan + Cyclophosphamide + ATG	1 (3.45)
Fludarabine + Busulfan + ATG	1 (3.45)
Cyclophosphamide + TBI	1 (3.45)
Prophylaxis for GVHD	
CSA + Methotrexate	26 (89.65)
CSA + Mycophenolate mofetil	2 (6.89)
Systemic immunosuppression	
PDN	13
CSA	1
Mycophenolate mofetil	1
PDN + CSA	9
PDN +tacrolimus	3

TBI: total body iradiation; ATG: anti-thymocyte globulin; GVHD: graft-*versus*-host disease; CSA: ciclosporin; PDN: prednisone.

In the present study, lichenoid lesions were the most frequent type of cGvHD lesions in the oral cavity (134 lesions in total), and they were mostly affecting the oral mucosa. Erythematous lesions and ulcers were less frequent (46 and 34 lesions in total, respectively) and the most affected site was also the oral mucosa. Some patients presented concomitant types of lesions and, in some cases, in different (Table 2). In the vermilion of the upper and lower lip, lichenoid lesions were the most frequent type of lesions (71% in total, table 2 and table 3).

**Table 2:** Distributions of simple and percentage frequencies related to the site of lesion

Sites	Lichenoid lesions n (%)	Erythema n (%)	Ulcer n (%)
Left oral mucosa	36 (26.9)	12 (26.1)	7 (20.6)
Right oral mucosa	31 (23.1)	13 (28.3)	8 (23.5)
Dorsum of the tongue	12 (9.0)	1 (2.2)	4 (11.8)
Right side of the tongue	12 (9.0)	5 (10.9)	4 (11.8)
Left side of the tongue	11 (8.2)	2 (4.3)	6 (17.6)
Upper lip mucosa	9 (6.7)	1 (2.0)	-
Soft palate	9 (6.7)	7 (15.2)	2 (5.9)
Lower lip mucosa	8 (6.0)	1 (2.2)	1 (2.9)
Hard palate	4 (3.0)	3 (6.5)	-
Ventral tongue	2 (1.5)	1 (2.2)	2 (5.9)
Total	134	46	34

**Table 3:** Type of lesion on vermilion of the lips

Sites	Lichenoid lesions n (%)	Erythema n (%)	Ulcer n (%)
Lower lip	12 (54.5)	2 (50.0)	4 (80.0)
Upper lip	10 (45.5)	2 (50.0)	1 (20.0)
Total	22	4	5

The analysis of lesion healing time considered the period between the first and the last day of the topical treatment (when the patient presented total regression of the lesion). The duration of injury was counted in days. The healing time of each type of lesion, according to the drugs used and their combinations, showed no significant difference in intragroup evaluations. However, lichenoid lesions responded better to dexamethasone-tacrolimus treatment, and erythema and ulcers responded better to dexamethasone in combination with systemic treatment (Table 4). Lichenoid lesions had a longer median healing time (105 days for total healing), whereas ulcers healed faster. It was not possible to perform statistical analysis for lesions of the vermilion of the upper and lower lip due to the small number of lesions observed in the patients.

**Table 4:** Intragroup comparison of healing time (in days) by type of lesion and drugs used during treatment

Type of lesion and drug used	Median	Minimum	Maximum	p value
Lichenoid lesions // Dexamethasone+ Systemic immunosuppression	105	14	539	0.232
Lichenoid lesions // Dexamethasone+ Tacrolimus + Systemic immunosuppression	91	35	427	
Lichenoid lesions // Dexamethasone	182	84	392	
Lichenoid lesions // Dexamethasone + Tacrolimus	84	28	252	
Lichenoid lesions // Tacrolimus	168	154	182	
Erythema // Dexamethasone + Systemic immunosuppression	42	21	420	0.467
Erythema // Dexamethasone +Tacrolimus + Systemic immunosuppression	84	21	147	
Erythema // Dexamethasone	140	7	280	
Erythema // Tacrolimus + Systemic immunosuppression	-	-	-	
Erythema // Dexamethasone + Tacrolimus	-	-	-	
Erythema // Tacrolimus	-	-	-	
Ulcer // Dexamethasone + Systemic immunosuppression	42	7	287	0.132
Ulcer // Dexamethasone + Tacrolimus + Systemic immunosuppression	77	63	84	
Ulcer // Dexamethasone	21	14	56	
Ulcer // Dexamethasone + Tacrolimus	*	*	*	
Ulcer // Tacrolimus + Systemic immunosuppression	*	*	*	
Ulcer // Tacrolimus	*	*	*	

* no cases.

When comparing lichenoid lesions with erythema and ulcers, we observed that the latter responded significantly better when treated with dexamethasone plus systemic treatment (p=0.047 and p=0.012, respectively), with no significant difference between ulcers and erythema. When patients were treated with dexamethasone combined with tacrolimus and systemic treatment, erythema and ulcers responded significantly better than lichenoid lesions (p=0.009 and p=0.000, respectively). In patients who used dexamethasone alone, ulcers responded significantly better than lichenoid lesions (p=0.017) (Table 5).

**Table 5:** Healing time in patients who used dexamethasone and systemic immunosuppression; dexamethasone, tacrolimus and systemic immunosuppression; and only dexamethasone

Patients who used dexamethasone and systemic immunosuppression				

Measures of position and measures of variation	Lichenoid lesions	Erythema	Ulcer	p value
Median	105	42	42	0.013*
Minimum	14	21	7	
Maximum	539	420	287	

* significance level 5%. Lichenoid lesions *versus* erythema – p=0.047; Lichenoid lesions *versus* ulcer – p=0.012; Erythema *versus* ulcer – p=0.101 - not significant.

**Table:** 

Patients who used dexamethasone, tacrolimus and systemic immunosuppression				

Measures of position and measures of variation	Lichenoid lesions	Erythema	Ulcer	p value
Median	91	84	77	0.011*
Minimum	35	21	63	
Maximum	427	147	84	

* significance level 1%. Lichenoid lesions *versus* erythema – p=0.009; Lichenoid lesions *versus* ulcer – p=0.000; Erythema *versus* ulcer – p=0.106 – not significant.

**Table:** 

Patients who used only dexamethasone for immunosuppression				

Measures of position and measures of variation	Lichenoid lesions	Erythema	Ulcer	p value
Median	182	140	21	0.042**
Minimum	84	7	14	
Maximum	392	280	56	

** significance level 1%. Lichenoid lesions *versus* erythema - p=0.196 not significant; Lichenoid lesions *versus* ulcer – p=0.017; Erythema *versus* ulcer – p=0.101 - not significant.

Although this was not the main objective of the study, we also collected data from median sialometry, which was 0.5mL (minimum=0mL/maximum=0.82mL) at the beginning, and 0.2mL (minimum=0mL//maximum=1.6mL) at the end of treatment. We observed hyposalivation in 13% of patients in the first examination, and in 30% of patients in the last examination. The mean salivary pH at the beginning and at the end of treatment was 6.5 (standard deviation (SD) =0.56) and 6.9 (SD=0.57), respectively. We found no association between hyposalivation/pH and response to treatment of oral cGVHD.

## DISCUSSION

In the present study, we observed that the use of topical medication was extremely important to obtain a positive response for all lesions, especially when combined with systemic treatment. In our center, dexamethasone is routinely used as the first choice of topical medication to treat oral cGvHD, and tacrolimus is used as a treatment enhancer, alone or in combination with dexamethasone, when the lesion does not present a good response to dexamethasone alone. In some situations, tacrolimus was not associated with dexamethasone due to lack of it, or when the patient did not accepted its use. During treatment for oral cGvHD, none of the study patients underwent treatment with extracorporeal photopheresis.

Therapy for oral cGvHD includes the use of corticosteroids, calcineurin inhibitors and analgesics. Topical mouthwash treatments are widely used due to their easy application and efficiency, but ointments are also effective in isolated lesions.^(13)^

Dexamethasone has been shown to be effective in treating oral lesions, considerably improving lesion severity, oral mucosa sensitivity and lichenoid lesions.^(14-16)^

Wolff et al.,^(16)^ performed a retrospective analysis of the use of dexamethasone along with prophylactic antifungal medication in 16 patients who had oral chronic GvHD lesions. Of these patients, nine presented total regression of the lesions, two presented partial regression and five did not respond to medication.

Tacrolimus has been frequently used in patients with cutaneous cGvHD, and results showed 72% of patients treated with this medication improved within a short period of time.^(17)^ Although tacrolimus has no current gel or ointment formulation, which makes its topical application to moist mucosa more difficult, case reports of topical treatment of oral cGvHD with the combination of tacrolimus and corticosteroids demonstrate improvement of the lesions, and total regression in up to one year of treatment.^(18)^

Some studies have shown that the use of topical tacrolimus in the oral cavity as an adjuvant treatment significantly improves patient quality of life, and decreases pain without systemic alterations to the patient.^(9)^ Lesions tend to improve when dexamethasone is combined with tacrolimus, achieving total regression or stabilizing without worsening.^(11)^

In our study, patients were only treated with tacrolimus after failing dexamethasone treatment, except for those with lip vermillion lesions. In the analysis of median healing time in patients grouped by the type of lesion, we observed better results when erythema and ulcers were treated with dexamethasone combined with systemic treatment, and when lichenoid lesions were treated with dexamethasone and tacrolimus, but the differences observed were not significant. When we compared median healing time between different types of lesions, we observed that erythema and ulcers responded better to treatment than lichenoid lesions.

According to Filipovich et al.,^(3)^ patients with moderate cGvHD can receive only topical therapy, unless this condition affects three or more organs, or a single organ presents a cGvHD score ≥2. This suggests that, if the patient has already completed a systemic medication protocol, topical treatment would be sufficient to treat oral lesions and maintain patient´s quality of life, while simultaneously bringing local benefits, without the need to resume the use of systemic treatment.

Mawardi et al.,^(11)^ reported the use of dexamethasone combined with tacrolimus, but in a shorter period of time compared to this study. Their findings were consistent with ours regarding erythematous and lichenoid lesions, and relief of pain.

Another treatment option for oral lesions is the use of clobetasol, which was not evaluated in this study, but demonstrates positive results similar to those reported for dexamethasone. Noce et al.,^(10)^ reported both medications had positive results; however clobetasol was more efficient in providing regression of symptoms and lesion healing.

Signs of cGvHD in the salivary glands were very frequent in our patients. We observed a 17% increase in the frequency of patients with hyposalivation after treatment initiation, despite the improvement of oral cGvHD. This observation corroborates the results observed by Imanguli et al.,^(19)^who reported that the compromised salivary gland and oral mucosal appear to be independent signs of oral cGvHD.

We had some limitations in the present study. The main limitation was the small number of patients included, leading to the existence of confounding variables (*e.g.,* absence of some types of treatment) during statistical analysis. In addition, we did not perform pain scale assessments. There are only a few studies evaluating the use of dexamethasone and tacrolimus, mostly case reports. Therefore, available evidence is still scarce, which limits the comparison of our results, and highlights the need to expand the investigations on topical treatment for oral cGvHD lesions. The assessment of the NIH oral cavity by scores provides an overall view of the mouth, but does not assess which type of lesion has regressed and what impact it has on quality of life. We must consider that ulcers cause pain and reduced quality of life, whereas lichenoid lesions do not normally cause pain. Therefore, an assessment of oral cGvHD should be included in clinical practice that reports the evolution of each type of lesions and not as a whole, depending on the impact on quality of life.

## CONCLUSION

In conclusion, our study shows that lichenoid lesions require more time to heal than other lesion types. Notably, lichenoid lesions tend to respond better to dexamethasone combined with tacrolimus, whereas erythema and ulcers respond better to dexamethasone combined with systemic treatment.
