# Supplementary material for: Treatment of oral chronic graft-versus-host disease: a retrospective cohort study
Source: Einstein (Sao Paulo). 2021 Oct 20;19:eAO6177. doi: 10.31744/einstein_journal/2021AO6177 (PMC8528385; doi:10.31744/einstein_journal/2021AO6177)
Supplement: Translation

# Translation: Tratamento da doença do enxerto contra hospedeiro crônica oral: um estudo de coorte retrospectivo

**Authors:** Gabriela de Assis Ramos, Taísa Domingues Boehmer Leite, Camila Brandão Lobo, Paulo Sérgio da Silva Santos, Maria Claudia Rodrigues Moreira, Héliton Spindola Antunes

**Affiliations:** 1 Brasil Divisão de Pesquisa Clínica, Instituto Nacional de Câncer - INCA, Rio de Janeiro, RJ, Brasil.; 2 Brasil Faculdade de Odontologia de Bauru, Universidade de São Paulo, Bauru, SP, Brasil.; 3 Brasil Centro de Transplante de Medula Óssea, Instituto Nacional de Câncer - INCA, Rio de Janeiro, RJ, Brasil.

**Keywords:** Doença do enxerto-hospedeiro, Transplante de medula óssea, Transplante de células tronco hematopoiéticas, Dexametasona, Tacrolimo

## Abstract

**Objetivo:**

Avaliar os pacientes com resposta completa da doença do enxerto contra hospedeiro crônica oral ao tratamento com imunossupressor.

**Métodos:**

Vinte e nove pacientes submetidos ao transplante alogênico de células tronco hematopoiéticas, com doença do enxerto contra hospedeiro crônica oral, foram incluídos neste estudo retrospectivo, de setembro de 2012 a fevereiro de 2018. Os pacientes foram tratados com dexametasona para bochecho associada ao tacrolimo pomada, dexametasona para bochecho associada ao tacrolimo tópico, tratamento imunossupressor sistêmico, e dexametasona tópica apenas.

**Resultados:**

O tempo médio para resposta completa das lesões liquenoides, eritema e ulcerações usando dexametasona e imunossupressor sistêmico foi de 105, 42 e 42 dias, respectivamente (p=0,013). Quando a dexametasona estava associada ao tacrolimo e a medicação imunossupressora sistêmica, o tempo médio para resposta completa das lesões liquenóides, eritema e ulcerações foi de 91, 84 e 77 dias (p=0,011). Quando foi utilizada apenas dexametasona, o tempo médio para resposta completa das lesões liquenoides, eritema e ulcerações foi de 182, 140 e 21 dias, respectivamente (p=0,042).

**Conclusão:**

Nosso estudo mostra que as lesões liquenoides requerem mais tempo para cicatrização completa. É notável que as lesões liquenoides tendem a responder melhor ao tratamento da dexametasona combinada com o tacrolimo e o imunossupressor sistêmico. Já o eritema e as ulcerações respondem melhor à dexametasona combinada com medicação imunossupressora sistêmica, e dexametasona apenas, respectivamente.

## INTRODUÇÃO

A doença do enxerto contra o hospedeiro crônica (DECH-c) é uma das principais complicações do transplante de células-tronco hematopoiéticas (TCTH). A doença afeta de 30% a 70% desses pacientes, causando respostas inflamatórias em diferentes órgãos e tecidos que se assemelham às de doenças autoimunes.^(1)^Na cavidade oral, os sintomas clínicos da DECH-c acometem principalmente a mucosa e a língua, caracterizando-se pela presença de lesões liquenoides na mucosa oral (estrias brancas), reconhecidas pelo consenso do *National Institutes of Health* (NIH) como lesões diagnósticas. Os pacientes também podem apresentar manifestações clínicas distintas que, isoladamente, não são suficientes para estabelecer o diagnóstico, como xerostomia, mucocele, atrofia da mucosa, pseudomembranas e úlceras, e outras manifestações clínicas comuns à DECH aguda e crônica, como gengivite, eritema, úlceras e dor.^(1-4)^

O último consenso do NIH^(5)^ recomenda tratamento tópico com corticosteroides de alta potência, inibidores da calcineurina e analgésicos.^(6,7)^ Os medicamentos tópicos locais têm o benefício de intensificar o tratamento, reduzindo ou eliminando os sintomas sem aumentar a imunossupressão sistêmica.^(8,9)^ Tratamentos tópicos podem fornecer melhores benefícios locais do que a terapia sistêmica sozinha.^(5)^

Apesar da ausência de evidências fortes, alguns estudos mostram que a maioria dos pacientes com DECH-c oral em uso de dexametasona combinada com tacrolimo responde positivamente ao tratamento.^(9,10)^ No presente estudo observacional, procuramos avaliar pacientes com DECH-c oral com resposta completa ao tratamento com imunossupressão tópica, associada ou não à imunossupressão sistêmica.

## OBJETIVO

Avaliar os pacientes com resposta completa da doença do enxerto contra hospedeiro crônica oral ao tratamento com imunossupressor.

## MÉTODOS

### Pacientes

Este estudo de coorte retrospectiva incluiu pacientes submetidos ao TCTH alogênico, de setembro de 2012 a fevereiro de 2018, registrados no Instituto Nacional de Câncer (INCA), Brasil. Foram considerados pacientes elegíveis homens e mulheres com idade ≥10 anos, que assinaram o termo de consentimento livre e esclarecido (TCLE), submetidos ao TCTH alogênico com diagnóstico de DECH-c oral (lesões liquenoides, lesões eritematosas e úlceras), que haviam recebido tratamento tópico com dexametasona com ou sem tacrolimo, com ou sem imunossupressão sistêmica, de acordo com a rotina hospitalar. Pacientes que faltaram à consulta, pacientes com resposta incompleta, e pacientes com diagnóstico de segundo tumor primário maligno na cavidade oral, orofaringe, nasofaringe, hipofaringe, laringe e glândulas salivares maiores foram considerados inelegíveis. Os dados foram coletados dos prontuários médicos e odontológicos dos pacientes. O estudo foi aprovado pelo Comitê de Ética em Pesquisa número do parecer 4606137; CAAE: 04066512.1.1001.5274.

### Tratamento tópico para doença do enxerto contra hospedeiro crônica oral

Os pacientes incluídos no estudo usaram, como primeira linha de tratamento, dexametasona para bochecho 0,1mg/mL, por três minutos, quatro vezes ao dia, após a higiene oral. Tacrolimo pomada 0,1% foi aplicado no local da lesão na cavidade oral duas vezes ao dia.^(11)^ A opção pelo tratamento tópico foi uma decisão individual do profissional com base na gravidade da doença e na disponibilidade da medicação.

### Tratamento sistêmico para doença do enxerto contra hospedeiro crônica

Os pacientes incluídos no estudo estavam usando os seguintes imunossupressores sistêmicos: prednisona (PDN), ciclosporina (CSA), tacrolimo, metotrexato (MTX) e micofenolato de mofetila (MFM), sozinhos ou em combinação.

### Avaliação de fluxo e pH da saliva

Considerando a alta frequência de acometimento de glândulas salivares em pacientes com DECH-c oral, com implicações na qualidade e quantidade da saliva,^(12)^ pacientes submetidos ao TCTH fizeram sialometria não estimulada durante a consulta de retorno (rotina odontológica do nosso centro). Um fluxo salivar abaixo de 0,1mL/minutos foi considerado hipossalivação.^(13)^ Para avaliar o pH salivar foram utilizadas as tiras indicadoras de pH *Color Card* MQuant^®^, indicador universal da Merck.

### Avaliação da doença do enxerto contra hospedeiro crônica oral

De acordo com o atendimento de rotina, os pacientes foram submetidos a exame clínico odontológico a cada 21 dias, desde o diagnóstico de DECH-c oral até a regressão total das lesões liquenoides, eritema e úlceras. Os casos graves eram acompanhados semanalmente. Uma resposta completa na boca corresponde a resolução das manifestações clínicas reversíveis relacionadas à DECH-c oral.

Não usamos a Escala de Classificação da Mucosa Oral do NIH para avaliar a resposta clínica, porque não queríamos uma avaliação geral da boca, mas uma avaliação específica do eritema, das úlceras e lesões liquenoides. Os seguintes dados foram coletados: localização (mucosa oral direita e esquerda, vermelhão do lábio inferior e superior, mucosa do lábio inferior e superior, borda lateral direita e esquerda da língua, face ventral e dorsal da língua, palato mole e palato duro) e duração da DECH-c oral, tratamento tópico utilizado (dexametasona para bochecho e tacrolimo pomada), tempo de tratamento e tratamento imunossupressor sistêmico utilizado.

### Avaliação dos níveis séricos de tacrolimo

Desde o diagnóstico, todos os pacientes tratados com tacrolimo sistêmico foram submetidos a testes séricos rotineiramente. No entanto, a avaliação da absorção após o tratamento tópico intraoral não foi realizada devido à falta de padronização de dia e horário de coleta.

### Análise estatística

O desfecho do estudo foi a avaliação da DECH-c oral com resposta completa ao tratamento tópico com dexametasona, com ou sem tacrolimo, com ou sem imunossupressão sistêmica, por meio de amostragem por conveniência. Para as variáveis discretas (tipos de lesão, localização das lesões e medicação utilizada), avaliamos frequências simples e percentagem. Para as variáveis contínuas, usamos medianas. O teste não paramétrico de Kruskal-Wallis foi usado para comparar o tempo de cicatrização durante o tratamento (em dias) por tipo de lesão e medicação utilizada e, quando significantes, foram aplicadas comparações múltiplas para avaliar as diferenças.

Todas as análises foram realizadas no *software* SPSS, versão 20.0, considerando um nível de significância de 5% de probabilidade. Valores de p menores ou iguais a 0,05 foram considerados estatisticamente significantes.

Dados de pacientes com perda de acompanhamento e que não puderam ser avaliados quanto ao uso da medicação e resposta ao tratamento não foram incluídos na avaliação.

## RESULTADOS

Este estudo coletou dados de prontuários médicos de pacientes para avaliar a resposta completa da DECH-c oral à dexametasona, com ou sem tacrolimo, e com ou sem imunossupressão sistêmica. Foram triados 76 pacientes com DECH-c oral, mas como se tratava de um estudo observacional, 45 pacientes foram excluídos por insuficiência de dados já que faltaram às consultas. Vinte e nove pacientes foram incluídos e, como o objetivo era avaliar pacientes com resposta completa, dois pacientes foram excluídos por apresentarem lesões com resposta parcial, consideradas não resolvidas.

A doença de base mais comum foi leucemia mieloide aguda (34,48%), seguida de leucemia linfoblástica aguda (31,03%). A maioria dos doadores eram aparentados (82,75%) e o esquema de condicionamento mais utilizado foi ciclofosfamida + bussulfano (48,28%) (Tabela 1).

**Tabela 1:** Características demográficas e clínicas

Dados demográficos	n (%)
Sexo	
Masculino	16 (55,2)
Feminino	13 (44,8)
Doença de base	
Leucemia mieloide aguda	10 (34,48)
Leucemia linfoblástica aguda	9 (31,03)
Leucemia mieloide crônica	4 (13,79)
Síndrome mielodisplásica	3 (10,34)
Anemia aplásica	2 (6,89)
Linfoma não Hodgkin	1 (3,44)
Doador	
Aparentado	24 (82,75)
Não aparentado	5 (17,25)
Esquema de condicionamento	
Ciclofosfamida + Bussulfano	14 (48,28)
Ciclofosfamida + TBI + ATG	3 (10,34)
Bussulfano + Fludarabina	3 (10,34)
Ciclofosfamida + TBI	3 (10,34)
Ciclofosfamida + Fludarabina	1 (3,45)
Bussulfano + Melfalano	1 (3,45)
Ciclofosfamida	1 (3,45)
Bussulfano + Ciclofosfamida + ATG	1 (3,45)
Fludarabina + Bussulfano + ATG	1 (3,45)
Ciclofosfamida + TBI	1 (3,45)
Profilaxia para DECH	
CSA + Metotrexato	26 (89,65)
CSA + Micofenolato de mofetila	2 (6,89)
Imunossupressão sistêmica	
PDN	13
CSA	1
Micofenolato de mofetila	1
PDN + CSA	9
PDN + tacrolimo	3

TBI: irradiação de corpo inteiro; ATG: globulina antitimócito; DECH: doença do enxerto contra hospedeiro; CSA: ciclosporina; PDN: prednisona.

No presente estudo, lesões liquenoides foram o tipo mais frequente de lesão da DECH-c na cavidade oral (134 lesões no total), acometendo principalmente a mucosa oral. Lesões eritematosas e úlceras foram menos frequentes (46 e 34 lesões no total, respectivamente) e o local mais acometido por estes tipos de lesão também foi a mucosa oral. Alguns pacientes apresentaram, concomitantemente, lesões de tipos diferentes e, em alguns casos, em locais diversos (Tabela 2). No vermelhão dos lábios superior e inferior, as lesões liquenoides foram o tipo de lesão mais frequente (71% no total, tabela 2 e tabela 3).

**Tabela 2:** Distribuições de frequências simples e percentuais relacionadas ao local da lesão

Local	Lesões liquenoides n (%)	Eritema n (%)	Úlceras n (%)
Mucosa oral esquerda	36 (26,9)	12 (26,1)	7 (20,6)
Mucosa oral direita	31 (23,1)	13 (28,3)	8 (23,5)
Face dorsal da língua	12 (9,0)	1 (2,2)	4 (11,8)
Lado direito da língua	12 (9,0)	5 (10,9)	4 (11,8)
Lado esquerdo da língua	11 (8,2)	2 (4,3)	6 (17,6)
Mucosa do lábio superior	9 (6,7)	1 (2,0)	-
Palato mole	9 (6,7)	7 (15,2)	2 (5,9)
Mucosa do lábio inferior	8 (6,0)	1 (2,2)	1 (2,9)
Palato duro	4 (3,0)	3 (6,5)	-
Face ventral da língua	2 (1,5)	1 (2,2)	2 (5,9)
Total	134	46	34

**Tabela 3:** Tipo de lesão no vermelhão dos lábios

Local	Lesões liquenoides n (%)	Eritema n (%)	Úlceras n (%)
Lábio inferior	12 (54,5)	2 (50,0)	4 (80,0)
Lábio superior	10 (45,5)	2 (50,0)	1 (20,0)
Total	22	4	5

A análise do tempo de cicatrização da lesão considerou o período entre o primeiro e o último dia de tratamento tópico (quando o paciente apresentou regressão total da lesão). A duração da lesão foi contada em dias. O tempo de cicatrização de cada tipo de lesão, de acordo com os medicamentos usados e suas combinações, não apresentou diferença significativa nas avaliações intragrupo. No entanto, as lesões liquenoides responderam melhor ao tratamento com dexametasona-tacrolimo, e o eritema e as úlceras responderam melhor à dexametasona combinada ao tratamento sistêmico (Tabela 4). As lesões liquenoides tiveram um tempo mediano de cicatrização mais longo (105 dias para a cicatrização total), enquanto as úlceras cicatrizaram mais rapidamente. Não foi possível realizar análise estatística para lesões do vermelhão do lábio superior e inferior devido ao pequeno número de lesões nesses locais em nossos pacientes.

**Tabela 4:** Comparação intragrupo do tempo de cicatrização (em dias) por tipo de lesão e medicação usada durante o tratamento

Tipo de lesão e medicação usada	Mediana	Mínimo	Máximo	Valor de p
Lesões liquenoides // Dexametasona + Imunossupressão sistêmica	105	14	539	0,232
Lesões liquenoides // Dexametasona + Tacrolimo + Imunossupressão sistêmica	91	35	427	
Lesões liquenoides // Dexametasona	182	84	392	
Lesões liquenoides // Dexametasona + Tacrolimo	84	28	252	
Lesões liquenoides // Tacrolimo	168	154	182	
Eritema // Dexametasona + Imunossupressão sistêmica	42	21	420	0,467
Eritema // Dexametasona + Tacrolimo + Imunossupressão sistêmica	84	21	147	
Eritema // Dexametasona	140	7	280	
Eritema // Tacrolimo + Imunossupressão sistêmica	-	-	-	
Eritema // Dexametasona + Tacrolimo	-	-	-	
Eritema // Tacrolimo	-	-	-	
Úlceras // Dexametasona + Imunossupressão sistêmica	42	7	287	0,132
Úlceras // Dexametasona + Tacrolimo + Imunossupressão sistêmica	77	63	84	
Úlceras // Dexametasona	21	14	56	
Úlceras // Dexametasona + Tacrolimo	*	*	*	
Úlceras // Tacrolimo + Imunossupressão sistêmica	*	*	*	
Úlceras // Tacrolimo	*	*	*	

* nenhum caso.

Ao comparar as lesões liquenoides com eritema e úlceras, observamos que estas últimas responderam significativamente melhor quando tratadas com dexametasona associada ao tratamento sistêmico (p=0,047 e p=0,012, respectivamente), sem diferença significante entre úlceras e eritema. Quando os pacientes foram tratados com dexametasona combinada com tacrolimo e tratamento sistêmico, o eritema e as úlceras responderam significativamente melhor do que as lesões liquenoides (p=0,009 e p=0,000, respectivamente). Em pacientes que usaram apenas dexametasona, as úlceras responderam significativamente melhor do que as lesões liquenoides (p=0,017) (Tabela 5).

**Tabela 5:** Tempo de cicatrização nos pacientes que usaram dexametasona e imunossupressão sistêmica; dexametasona, tacrolimo e imunossupressão sistêmica; e dexametasona apenas

Pacientes que usaram dexametasona e imunossupressão sistêmica				

Medidas de posição e medidas de variação	Lesões liquenoides	Eritema	Úlceras	Valor de p
Mediana	105	42	42	0,013*
Mínimo	14	21	7	
Máximo	539	420	287	

* nível de significância 5%. Lesões liquenoides *versus* eritema – p=0,047; Lesões liquenoides *versus* úlceras – p= 0,012; Eritema *versus* úlceras – p=0,101 – não significativo.

**Table:** 

Pacientes que usaram dexametasona, tacrolimo e imunossupressão sistêmica				

Medidas de posição e medidas de variação	Lesões liquenoides	Eritema	Úlceras	Valor de p
Mediana	91	84	77	0,011*
Mínimo	35	21	63	
Máximo	427	147	84	

* nível de significância 1%. Lesões liquenoides *versus* eritema – p=0,009; Lesões liquenoides *versus* úlceras – p=0,000; Eritema *versus* úlceras – p=0,106 – não significativo.

**Table:** 

Pacientes que fizeram imunossupressão com dexametasona apenas				

Medidas de posição e medidas de variação	Lesões liquenoides	Eritema	Úlceras	Valor de p
Mediana	182	140	21	0,042**
Mínimo	84	7	14	
Máximo	392	280	56	

** nível de significância 1%. Lesões liquenoides *versus* eritema – p=0,196 não significativo; Lesões liquenoides *versus* úlceras – p=0,017; Eritema *versus* úlceras – p=0,101 – não significativo.

Embora este não tenha sido o objetivo principal do estudo, também coletamos dados de sialometria mediana, que foi de 0,5mL (mínimo=0mL; máximo=0,82mL) no início, e 0,2mL (mínimo=0mL; máximo=1,6mL) no final do tratamento. Observamos hipossalivação em 13% dos pacientes no primeiro exame, e em 30% dos pacientes no último exame. O pH médio da saliva no início e no final do tratamento foi de 6,5 (desvio padrão (DP) =0,56) e 6,9 (DP=0,57), respectivamente. Não encontramos associação entre hipossalivação/pH e resposta ao tratamento de DECH-c oral.

## DISCUSSÃO

No presente estudo, observamos que o uso de medicação tópica foi de extrema importância para a obtenção de resposta positiva em todas as lesões, principalmente quando associada ao tratamento sistêmico. Em nosso centro, a dexametasona é usada rotineiramente como medicação tópica de escolha para o tratamento de DECH-c oral, e o tacrolimo é usado como potencializador do tratamento, sozinho ou em combinação com a dexametasona, quando a lesão não apresenta boa resposta à dexametasona sozinha. Em algumas situações, o tacrolimo não foi associado à dexametasona por questões de indisponibilidade, ou quando o paciente não aceitou seu uso. Nenhum dos pacientes do estudo foi submetido a tratamento com fotoferese extracorpórea durante o tratamento para DECH-c oral.

O tratamento da DECH-c oral inclui o uso de corticosteroides, inibidores da calcineurina e analgésicos. Tratamentos com bochecho tópico são amplamente utilizados devido à sua fácil aplicação e eficiência, mas pomadas também são efetivas em lesões isoladas.^(13)^

A dexametasona demonstrou ser efetiva no tratamento de lesões orais, melhorando consideravelmente a gravidade da lesão, a sensibilidade da mucosa oral e as lesões liquenoides.^(14-16)^

Wolff et al.,^(16)^ realizaram uma análise retrospectiva do uso de dexametasona em combinação com medicação antifúngica profilática em 16 pacientes com lesões de DECH-c oral. Desses pacientes, nove apresentaram regressão total das lesões, dois apresentaram regressão parcial e cinco não responderam à medicação.

O tacrolimo tem sido frequentemente usado em pacientes com DECH-c cutâneo, e os resultados demonstram que 72% dos pacientes tratados com este medicamento melhoraram em um curto período de tempo.^(17)^ Embora o tacrolimo não tenha, atualmente, formulação em gel ou pomada, o que dificulta sua aplicação tópica na mucosa úmida, relatos de casos de tratamento tópico de DECH-c oral com a combinação de tacrolimo e corticosteroides demonstram melhora das lesões, e regressão total em até um ano de tratamento.^(18)^

Alguns estudos demonstraram que o uso de tacrolimo tópico como tratamento adjuvante na cavidade oral melhora a qualidade de vida do paciente de forma significativa, e diminui a dor sem alterações sistêmicas ao paciente.^(9)^ As lesões costumam melhorar quando a dexametasona é combinada com tacrolimo, alcançando regressão total ou estabilizando sem piora.^(11)^

Em nosso estudo, os pacientes foram tratados com tacrolimo apenas após falha do tratamento com dexametasona, exceto aqueles com lesões no vermelhão dos lábios. Na análise da mediana do tempo de cicatrização dos pacientes agrupados por tipo de lesão, observamos melhores resultados quando o eritema e as úlceras foram tratados com dexametasona associada ao tratamento sistêmico, e quando as lesões liquenoides foram tratadas com dexametasona e tacrolimo, mas as diferenças observadas não foram significativas. Quando comparamos o tempo de cicatrização mediano entre os diferentes tipos de lesões, observamos que o eritema e as úlceras responderam melhor ao tratamento do que as lesões liquenoides.

Segundo Filipovich et al.,^(3)^ os pacientes com DECH-c moderada podem receber apenas tratamento tópico, a menos que a doença acometa três ou mais órgãos ou quando um único órgão apresente um escore de DECH-c ≥2. Isso sugere que, caso o paciente já tenha finalizado um protocolo de medicação sistêmica, o tratamento tópico seria suficiente para tratar as lesões orais e manter a qualidade de vida do paciente, ao mesmo tempo em que traria benefícios locais, sem a necessidade de reiniciar o tratamento sistêmico.

Mawardi et al.,^(11)^ estudaram o uso de dexametasona associada ao tacrolimo, mas com duração mais curta do que a deste estudo. Seus achados foram consistentes com os nossos no que tange às lesões eritematosas e liquenoides, e ao alívio da dor.

Outra opção de tratamento para lesões orais é o uso de clobetasol que não foi avaliado neste estudo, mas apresenta resultados positivos semelhantes aos relatados para dexametasona. Noce et al.,^(10)^ relataram que os dois medicamentos apresentaram resultados positivos; porém o clobetasol foi mais eficiente em proporcionar regressão de sintomas e cicatrização das lesões.

Sinais da DECH-c nas glândulas salivares foram vistos com muita frequência em nossos pacientes. Observamos um aumento de 17% na frequência de pacientes com hipossalivação após o início do tratamento, apesar da melhora da DECH-c oral. Esta observação corrobora os resultados observados por Imanguli et al.,^(19)^ que relataram que o comprometimento da mucosa oral e glândulas salivares parecem ser sinais independentes de DECH-c oral.

Nosso estudo teve algumas limitações. A principal delas foi o pequeno número de pacientes incluídos, levando à existência de variáveis de confusão (por exemplo, ausência de alguns tipos de tratamento) durante a análise estatística. Além disso, não foram realizadas avaliações com escalas de dor. Existem poucos estudos avaliando o uso de dexametasona e tacrolimo, a maior parte deles sendo relatos de casos. Portanto, as evidências disponíveis ainda são escassas, o que limita a comparação de nossos resultados, e destaca a necessidade de expandir as investigações sobre o tratamento tópico das lesões da DECH-c oral. A avaliação da cavidade oral do NIH por escores fornece uma visão geral da boca, mas não avalia que tipo de lesão regrediu e o impacto na qualidade de vida. Devemos considerar que as úlceras causam dor e redução da qualidade de vida, ao passo que as lesões liquenoides normalmente não causam dor. Portanto, é importante incluir, na prática clínica, uma avaliação da DECH-c oral que relate a evolução de cada tipo de lesão, e não apenas uma avaliação geral, dependendo de seu impacto na qualidade de vida.

## CONCLUSÃO

Concluindo, nosso estudo mostra que as lesões liquenoides requerem mais tempo para cicatrizar do que outros tipos de lesão. Notavelmente, lesões liquenoides tendem a responder melhor ao tratamento com dexametasona associada ao tacrolimo, enquanto o eritema e as úlceras respondem melhor à dexametasona combinada ao tratamento sistêmico.
